# The complexities of the diet-microbiome relationship: advances and perspectives

**DOI:** 10.1186/s13073-020-00813-7

**Published:** 2021-01-20

**Authors:** Emily R. Leeming, Panayiotis Louca, Rachel Gibson, Cristina Menni, Tim D. Spector, Caroline I. Le Roy

**Affiliations:** 1 The Department of Twin Research, St Thomas’ Hospital, King’s College London, 3-4th Floor South Wing Block D, Westminster Bridge Road, London, SE1 7EH UK; 2 Department of Nutritional Sciences, King’s College London, Franklin-Wilkins Building, 150 Stamford Street, London, SE1 9NH UK

**Keywords:** Personalised nutrition, Gut microbiome, Diet, Research methods

## Abstract

Personalised dietary modulation of the gut microbiota may be key to disease management. Current investigations provide a broad understanding of the impact of diet on the composition and activity of the gut microbiota, yet detailed knowledge in applying diet as an actionable tool remains limited. Further to the relative novelty of the field, approaches are yet to be standardised and extremely heterogeneous research outcomes have ensued. This may be related to confounders associated with complexities in capturing an accurate representation of both diet and the gut microbiota. This review discusses the intricacies and current methodologies of diet-microbial relations, the implications and limitations of these investigative approaches, and future considerations that may assist in accelerating applications. New investigations should consider improved collection of dietary data, further characterisation of mechanistic interactions, and an increased focus on -omic technologies such as metabolomics to describe the bacterial and metabolic activity of food degradation, together with its crosstalk with the host. Furthermore, clinical evidence with health outcomes is required before therapeutic dietary strategies for microbial amelioration can be made. The potential to reach detailed understanding of diet-microbiota relations may depend on re-evaluation, progression, and unification of research methodologies, which consider the complexities of these interactions.

## Background

The field of microbiome research has progressed rapidly in recent years, driven by technological advances and reduced costs of analysis [1]. Significant insights have been made into the nature of microbial communities and their impact on host health [1]. However, there are inherent challenges and complexities in defining the boundary of a ‘healthy’ microbiome landscape [2]. Microbial signatures are highly individual and multi-dimensional [3] with multiple landscapes likely to be considered healthful depending on the context [4]. In a study by Ghosh et al., researchers investigated the impact of a 1-year Mediterranean diet intervention on the gut microbiota and frailty [5]. The authors remind us of the Anna Karenina principle which posits that healthy individuals typically display microbiomes more similar to one another, while those of unhealthy individuals are each aberrant in their own way [5, 6]. The involvement of the microbiome in an extensive number of diseases suggests the need for its incorporation into contemporary medicine for an improved understanding of disease pathogenesis and pathology [7]. A clear link exists between loss of keystone taxa that drive microbiome structure and function (such as *Faecalibacterium prausnitzii* [8]), and various disease states [9]. However, one of the biggest challenges in microbiome research is discerning association from causation [10]. To date, there is limited evidence to support causation in humans, predominantly due to limitations in accurately manipulating the human microbiome [11]. Methods such as faecal microbiome transplants (FMT) have provided evidence that the microbiome alone can causally alter the human phenotype [12]. The ability of FMT from lean donors to reorientate host glucose metabolism is influenced by the recipient’s baseline microbial profile [13]. This could be explained, in part, by species from both recipient and donors remaining durably in the gut post-FMT, demonstrating the difficulties in precisely manipulating the composition of the microbiome [14].

Besides invasive solutions, therapeutic modulation of the gut microbiota could be achieved through diet [15]. Human and animal models have highlighted the influence of diet in shaping the gut microbial community through the provision of substrates for the metabolic requirements of individual or subsets of microbial taxa [16], in addition to modulating host gut microbiota crosstalk [17]. Although diet provides one of the most promising means of selectively altering the microbiome [18], current descriptions of human dietary habits and food compositions provide a simplistic insight into a complex world that is still largely unmapped [19]. Homogenous outcomes within the nutrition field are stymied by several factors. An accurate description of dietary intake is fundamental to health and nutrition research, yet capturing dietary exposure is challenging [20]. As per the microbiome, one’s diet is often composed of a multitude of components that are poorly characterised individually and rarely investigated in combination or as a food matrix structure [15, 21]. While current investigations into the diet-microbial relationship have provided a broad understanding of some of these relations [22], further progress has been restricted.

We propose this review as a readout of the current flaws in diet-microbiome studies while proposing points of improvements that should enable the furthering of current knowledge in this field. Together, this should pave the way towards a global improvement of population health. Firstly, we describe the gut microbiome as a complex ecosystem with multiple interactions, secondly, we discuss the intricacies of dietary investigations, and thirdly, we consider the importance of combining these two fields when researching diet-microbe relations. For the scope of this review, we refer to the microbiome as an ecosystem which incorporates all microorganisms together with the metabolites and other components of the gut environment as defined by Marchesi and Ravel [23]. However, the predominant focus of the research within this field has been towards investigating bacteria and their interactions.

## The gut microbiome a complex ecosystem

### Multiple intricate interactions

While phyla and functional pathways are widespread within the population (encountered in over 50% of individuals), species tend to be more subject-specific with, on average, two unrelated individuals sharing approximately 43% of species [24–26]. Functional equivalence can be explained by the notion of niches with multiple species interacting in a competitive or synergistic nature [27]. Hence, past work has shown that manipulation of a single species can prove difficult [25], while the system-wide influence of the microbial community can be achieved [28, 29]. A number of studies reported the involvement of microbes in an orchestrated maintenance of host homeostasis. Microbial networks of species and metabolic products, for example, act on both microbes and host cell gene expression selectively dictating cellular productivity [7], emphasising the urgent development of tools that can capture the full complexity of these interactions [30, 31].

**Fig. 1:**
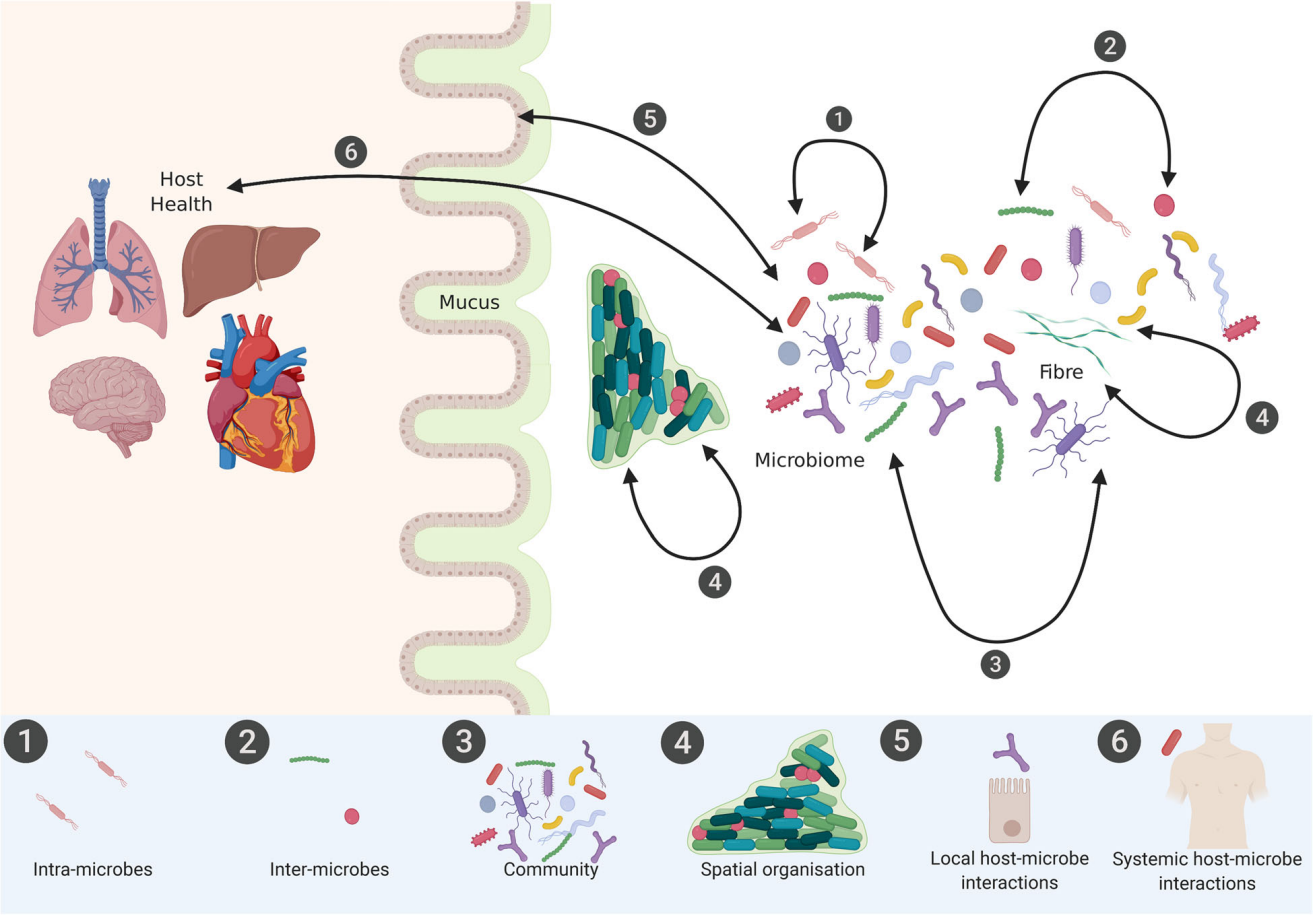
Understanding interactions between microbes, the microbiome, and the host both locally and systemically to enable its manipulation in order to improve human health. Suggested approaches for the characterisation of (1) intra-microbe interactions include in vitro mono- and co-culture systems; (2) inter-microbe interactions include in vitro co-culture and mass culture systems alongside quorum sensors to detect autoinducers that orchestrate collective behaviours [36, 37]; (3) communities include in vitro synthetic continuous communities with novel microarray technologies [38]; (4) spatial organisation include confocal microscopy integrated with multi-dimension algorithms alongside multi-omic technologies [39]; and (5) local host-microbe interactions include in vivo animal models accompanied by metabolomics providing a direct functional output of the metabolite profile, a result of local-host-microbial interactions [40], whereby the simultaneous profiling and integration of various -omic technologies is necessary to then identify (6) interactions at the molecular level systemically [40]. Image created with Biorender.com

Microbial species observed within the modern gut may have evolved through ecological adaptations of host-microbe interactions to ensure microbial stability in response to periods of limited nutritional availability [7]. Even so, the gut microbiome is not a static community. The complexity of studying the microbial ecosystem is deepened by its temporal dynamics, shifting diurnally, seasonally, and in constant flux [32, 33] requiring longitudinal investigations [34]. The high rate of strain-level turnover may allow for microbial evolution to impact certain species’ long-term persistence and colonisation in the gut [35]. In vitro and model organisms have assisted our current understanding of these dynamics, however through the lens of a reduced or simplified microbial ecosystem [36]. Mathematical models should be developed combining large human datasets together with in vitro and in vivo modelling in order to incorporate these dynamics (Fig. 1) [41]. Likewise, further emphasis must be made to interpret temporal dynamics through longitudinal sampling, with a limited number of longitudinal microbiome studies to date [27].

Mechanistic studies are difficult to implement in humans due to immense genetic and lifestyle heterogeneity together with ethical limitations [42]. Consequently, the functional contribution of the gut microbiome to human physiology remains largely unexplored [43]. Current knowledge stems from animal models, in vitro and in vivo assays, and is complemented by population-based studies [42]. In this way, Suez et al. displayed that non-caloric artificial sweeteners (NAS) induced glucose intolerance in both mice and humans via modulation of the gut microbial community [44]. Functional analysis of the saccharin-associated metagenome suggests a number of enriched pathways in heterocyclic compound metabolism, with a proliferation of certain taxa possibly linked with their capacity to harness saccharin as an energy source [44]. Several studies have explored mechanistic modelling directly in humans. Sanna et al. used bidirectional Mendelian randomisation (MR) analyses to assess causality in a cohort of 1539 individuals from the LifeLines cohort [45]. An increase in microbial butyrate production driven by host genetics was reported to be associated with an ameliorated insulin response to an oral glucose-tolerance challenge. Likewise, abnormal production or absorption of propionate was shown to be causally related with an increased risk of type II diabetes [45]. Characterising the functional differences of the microbial taxa is fundamental in understanding their impact on human physiology [43]. Functional -omics and FMT [46] have demonstrated their potential in assisting in the identification of functional gut microbial traits [43], in conjunction with other more traditional measures. Detailed theoretical simulations to characterise the functional difference of specific microbiological ecosystems are also thought to be feasible, such as Larsen and Claassen’s work which support the mechanistic link between alpha-diversity and health [47].

The spatial organisation of microbial communities is critical to understanding microbial signalling and metabolic interactions at a micron-scale but is yet relatively uncharted territory (Fig. 1) [48, 49]. Within this bio-geography, microbial taxa tend to be localised according to their functional niche, for example, anaerobic taxa typically residing to the interior and consumers and producers of a metabolite found to be within equidistance of each other [50, 51]. The availability of relevant substrates within the gut is also expected to drive this spatial organisation. While multi-omic techniques such as metagenomics, metabolomics, transcriptomics, and proteomics provide key tools in investigating the intricate crosstalk within the microbial community and between microbes and host, these techniques tend to be applied to homogenised samples [49] with spatial heterogeneity typically neglected. The use of confocal microscopy on biopsy samples enables the identification of single cells, a specific challenge in the dense cellular environment within the lower intestine [49]. Several tools have been developed to facilitate semi-automated curation of cell boundaries, which can be challenging due to close bacterial contact, or pixel-based quantitative measures for large-scale measurements [49]. Integration of spatial organisation within multi-dimensional algorithms, in conjunction with -omic technology, may assist in advancing the study of the organisation and dynamics of the microbial community and its relationship with host health. Retention of the structure by dissecting and spreading out the sample on a slide may aid in revealing the spatial organisation. With this method, investigations of the oral microbiome using dental plaque allowed for characterisation of the microbiota as highly structured with multi-genus consortia [50]. The gold standard approach is whole-stool homogenised sampling, although implementation of this can be impractical. Whether to collect complete or partial stool samples or biopsy specimens should be considered by the investigators and is dependent on feasibility, costs, patient cooperation, and downstream analysis [52].

Finally, the typical faecal sample represents the final point of a developing and maturing ecosystem through the gastrointestinal tract [53]. With varying gut transit times between individuals, the collection of dietary data and its corresponding stool sample can be fraught with inconsistencies [54]. Faecal consistency, a proxy for transit time, has been identified as a major co-variate of microbial structure. This suggests that transit time data should be included in future microbiome investigations and considered when capturing dietary information [55]. While there are several validated measures of transit time, including scintigraphy and radiopaque markers, many are expensive with high participant burden [56]. Other cheaper scalable measures include the blue dye method, faecal consistency, and frequency [57].

### Optimising microbial data collection, storage, and analysis

Microbiome data processing pipeline, collection, storage, and analysis of samples are particularly vulnerable to significant error [54, 58] that contribute to high variability in research outcomes [59]. Collection methods include variable storage temperatures, freeze-thaw cycles, lysis conditions, and physical perturbations [54]. While a detailed approach is required, protocols that are perceived as too arduous can induce attrition bias in and of themselves [54]. Numerous techniques and differing protocols have been suggested with a verified gold standard approach yet to be established [52].

Traditional culture-based technologies that have been used to investigate the microbial ecosystem have recently regained attention with the development of new methods enabling the culturing of an extended number of bacteria from the human gut. Though bacterial taxa including Ruminococcaceae and *Faecalibacterium* tend to be overrepresented by these methods [60]. Besides, only 50–60% of bacterial species present in the human gut have been observed to produce spores resistance to multiple environmental challenges [60], thereby facilitating transmission from host-to-host [61], consequently limiting the scope for FMT studies. While this is an expensive, cumbersome approach with clear methodological limitations, the majority of current knowledge within microbiome research originates from culture-based studies and has been informative in steering future directions with more progressive techniques [62].

Development of new methodologies should assist in addressing sample processing bias. For example, whole shotgun metagenomic sequencing has allowed investigators to bypass the PCR amplification used within 16s rRNA sequencing related to an overestimation of certain taxa [63]. Outside of the wet lab, a wide variety of bioinformatics tools can be used to classify microbial taxa from sequencing data [64]. A number of bioinformatic tools are publicly available for quality control, sequence assembly, operational taxonomic units, functional profiling, and prediction and to determine diversity evenness and richness [65]. Resources such as PICRUSt use evolutionary modelling to predict metagenomes from 16S data and a reference genome database [66] and have shown correlations between inferred and metagenomically measured content of close to 0.9 [66]. Platforms such as MGnify, a free-to-use platform for the assembly, analysis and archiving of microbial data have allowed for publicly available analysed datasets [67]; however, extensive action is required to populate these platforms, and differences between pipelines can also lead to variations in outcomes.

Improved accuracy and throughput of DNA sequencing techniques, together with multi-omic analysis and mechanistic experiments in animal models, increased our understanding on the structure and function of the microbiome in health and disease [42]. Several methodologies are required to further characterise how microbial functionality may relate to health and disease [68]. These include but are not limited to (i) the development and application of molecular and cellular high-throughput measurements; (ii) experimental models and human studies of direct molecular effects [43], for example, the use of germ-free mice can provide insights into disease causality [69]; and (iii) the incorporation of transcriptomics and epigenetic data into the gut metagenomic profile. These allow us to understand how a shift in microbiome composition can modify pathways involved in disease pathogenesis. For instance, microbiota-dependent histone modification has emerged as a molecular mechanism involved in tumour suppression, although findings are currently non-conclusive [34]. These steps will facilitate research in shifting away from inferences and towards a more causative understanding of the relationship between the microbiome and the pathogenesis of disease states.

## Capturing dietary diversity and food interactions

### Food is rarely consumed on its own and contains numerous compounds

The complexity of the human gut microbiome is largely mirrored by that of diet [4]. While we have a broad understanding of the impact of diet on the gut microbiota, formulating meaningful targeted dietary strategies remains a key challenge [70]. Foods are rarely consumed alone, and the number of available combinations is incalculable, although some may be more recurrent than others [19]. Diet is a highly individualised, multifaceted and changing measure, yet specifically linked to the geographical and cultural context, restraining research generalisation [71, 72]. Variable nutrition content within the same food item related to climate, soil type, and season also contribute confounding factors and limitations [73, 74]. Food composition values within food composition databases are typically obtained from laboratory analyses; however, due to the high procedural costs, many values are estimated based on conversion factors or as a ratio of similar food types [75]. There is limited potential to consider the biochemical digestibility, absorption and subsequent bioavailability of substrates for microbial communities as a time-dependent process and one which is highly variable between individuals [76]. No method of collecting dietary data is totally devoid of error, and the efficacy of each is dependent on the scenario. The guidance of research dietitians in diet investigations is strongly recommended, with inconsistent findings as a result of suboptimal use of dietary assessments. While numerous significant or strong observations have been determined by epidemiological studies, these have not always been supported by the outcomes of randomised controlled trials [77]. Failure to confirm a dietary effect may be due to a magnitude of cumulative biases [78], in conjunction with a small effect size, amongst others, rather than a lack of validity.

While a shift towards bigger datasets is undoubtedly required, this cannot be considered a ‘catch-all’ solution. With dietary bioactive compounds acting synergistically, and present within a multitude of food sources, almost all nutritional variables correlate between each other and health outcomes, particularly evident in large datasets [79]. Other factors such as eating behaviours, eating times, nutrient provenance, habitual diet, and other social and behavioural factors are not currently addressed within the majority of diet-microbiome investigations, yet all play a role in mediating host health [15, 80]. A move from simplistic reductionist strategies towards multi-faceted approaches is required.

Advancing nutritional research techniques has not progressed at the same pace as the rapid development of microbial investigations in the last decade. To improve understanding of diet-microbial relations, drastic progress is required to further our ability to characterise diet beyond the established macro and micronutrients. Emphasis on the importance of accurate dietary data methodologies and application of techniques typically applied within other scientific research fields, such as machine learning, may assist. While the nutritional community includes outstanding scientists, a large number of dietary research are undertaken by investigators in other fields, without the input of dietitians, nutritionists, or scientists trained in nutritional epidemiology. This may limit the quality of dietary data collection, processing, analysis, and reporting. For diet-microbial investigations, the involvement of a trained nutrition research professional should undoubtedly improve research outcomes and aid in the elucidation of some of the intricacies within these relations.

### Dietary data collection, processing, and analysis for microbiome studies

#### Study design

**Fig. 2:**
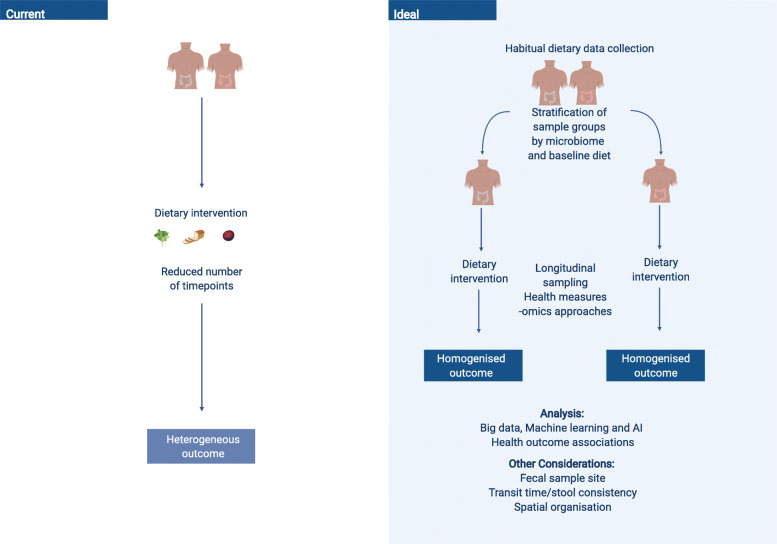
Current approaches vs. ideal approaches (image modified from Leeming et al. [15]). Current microbiome-diet-host approaches carry a number of caveats which may contribute to highly heterogeneous responses, such as the individualised microbiome [15]. A new ideal approach that may allow for further elucidation of diet-microbial-host relations includes stratification by microbial signature, collection of habitual diet data, longitudinal sampling and big data, machine learning, and AI approaches in order to enhance the predictability of outcomes in response to the dietary intervention. Image created with Biorender.com

In designing study protocols for diet-microbiome investigations, collaborations between dietitians and/or nutritionists and microbiologists, epidemiologists, and biostatisticians are essential in order to capture a broad spectrum of accurate data. Establishing the cause and effect of diet has been acknowledged as challenging [81]. Nutritional epidemiology typically identifies dietary components that modify health risk, which can then be tested within a clinical trial [82]. Controlled feeding studies are considered to be robust in determining cause-and-effect relationships between diet and physiological health outcomes, as they facilitate deep phenotypical analysis [81]. Nevertheless, only a small fraction of studies are of an experimental study design within the diet-microbial field. Interventions investigating diet and health require a large amount of participant burden, substantial time and financial costs, and a high level of participant commitment [83]. Habitual diet is acknowledged to play a strong role in shaping the microbial ecology through the daily provision of substrates [15]. The collection of habitual dietary data may be essential regardless of the investigative format and should be incorporated into experimental study designs (Fig. 2). Previous work suggested that gut microbial communities can be clustered into typical ‘enterotypes’, defined as densely populated multi-dimensional areas in the gut microbial community. These ‘enterotypes’ could be used as a way to stratify samples to reduce complexity [84] and previously have been linked to cardiometabolic risk [85], and a differential response to T2D treatment [86]. Microbial and metabolic phenotypes exhibit enterotype-specific links, emphasising the importance of enterotype stratification in investigating metabolic responses to diet [87]. Multi-centre studies allow for the investigation of enterotypes in wider population groups. These acknowledge the influence of geographical, ethnic, and cultural influences on the microbiome and diet amongst others. Investigations of diet-microbe relations, particularly multi-centre randomised control trials, that stratify according to enterotype profile and account for baseline habitual diet, with longitudinal sampling and health measures, may lead to increased homogeneity of outcomes. In combination, these suggestions would undoubtably assist in promoting general, and individualised or enterotype-based, diet-microbe therapeutic recommendations for the prevention or amelioration of relevant disease states (Fig. 2).

#### Measurement

Investigators of diet-microbiome relations often rely on food frequency questionnaires and self-reported food diaries [88]. Yet, multiple weighed 24-h dietary recalls, involving a retrospective assessment held by a trained nutrition professional or dietitian, are generally acknowledged to provide the highest accuracy in capturing dietary intake [72]. Resources such as the DIETary Assessment Tools NETwork (DIET@NET), who developed the Nutritools website [89], facilitate researchers’ awareness of the strengths and weaknesses of dietary assessment methods [90, 91], summarised in Table 1. High participant burden and costs, such as interview time and data entry, limit the utility of dietary recalls for large cohorts [72], though recent progress in technological applications, such as web-, app-, and computer-based 24-h dietary recall tools, may mitigate some of these limitations [93]. A minimum of four to eight repeated 24-h dietary recalls have been previously recommended to accurately characterise habitual dietary intake [94, 95]. Typically, food frequency questionnaires (FFQs) are employed in nutrition research for large population studies as a cost-effective tool for assessing habitual diet [88].

**Table 1:** Advantages and disadvantage of dietary assessment methods

Dietary assessment tool	Strengths	Weaknesses
**Retrospective**		
**Dietary recalls**—short-term method, where foods and drinks consumed are recalled.	- Self-reported manually or electronically. - Can be interview led, face-to-face, by phone, or online. - Typically, recall past 24 h but can be employed to recall longer durations/instances. - Multiple 24-h recalls can estimate habitual intakes. - Facilitates collection of extra information (meal timing, frequency, and location). - Flexibility of collected data is applicable to diverse research questions and analytical methods. - Limited literacy skills and cultural differences can be overcome using an interviewer. - Moderate participant burden and high compliance rates. - A skilled interviewer using multi-pass methods can prompt information, increasing accuracy.	- Habitual intake can change during the recall period. Overcome if participants are not forewarned. - Limited accuracy when recalling distant periods. - Unsuitable for subjects with memory disorders or elderly. - Items often omitted and incorrect items can be recalled. - One 24-h recall has limited accuracy, typically underestimating intake and overlooks day-to-day variation. - Moderate-to-high burden when analysing, requiring standardised protocols. - Expensive to employ face-to-face interviews in studies with large samples. - Reliance on subjects’ ability to remember portion size.
**Food frequency questionnaire**—retrospective methods recording frequencies of common foods over a period of time (weeks, months, years). Can be qualitative (frequency only), semi-qualitative (estimated portion size), or quantitative (portion size queried).	- Self-reported manually or electronically or interviewer led. - Useful for estimating long-term intakes retrospectively. - Low cost and participant burden, higher completion rate, applicable to large population studies. - Comprehensive questionnaires can estimate total nutrient intake if the portion size is prompted. - Can utilise short questionnaires specific to foods or nutrients pertinent to the research question. - Analysis is typically less burdensome on researchers.	- Arduous for participants if > 100 food items are queried. - Limits comparisons across cultures/countries unless comparable diets. - Typically, shorter questionnaires have less reliability and accuracy of intake. - Relies on participants’ memory, literacy, and numeracy skills. Longer periods of time reduces the accuracy of intakes. - Requires a proxy for accurate reporting in children. - Prone to misreporting, particularly with longer questionnaires. - Finite list of items included in the questionnaire. - Expensive software required to convert frequencies to nutrients.
**Prospective**		
**Food diaries**—prospective methods where details of everything consumed is logged over several days. Portions can be either estimated by the subject or via photographical evidence or weighed by the subject or research assistant at the time of consumption.	- Provides detailed depiction of foods and drinks consumed, including portions. - Generates good estimates of short-term dietary intake, **if conducted thoroughly**. - Facilitates collection of contextual data (meal timing, location, satiety levels). - Not influenced by subjects’ memory if recorded prospectively. - Weighed provides more accurate quantitative intake, can also include ingredients and food waste. - Can be conducted via digitally or manually. - Prompts can promote the inclusion of specific foods, nutrients or occasions, pertinent to the research question and limit misreporting. - Reasonably cost-effective. - Accuracy increases with standardised protocols and analysis.	- Not applicable to retrospective studies. - High participant burden, particularly over longer durations, adding to the high participant burden of microbiome research. - Costly in time and resources for coding and analysis. - Compliance rate reduces as the duration of recording increases. - Requires sufficient literacy and numeracy skills of subject/proxy. - Heavy reliance on subjects’ perception of portions (can be improved with photographs). - Relies on trust that the diary is complete at the time of consumption and not as a recall.
**Dietary checklist**—prospective short-term method where specified foodstuffs are ticked from a checklist over a number of days. Can include frequencies or portion sizes. Typically used as a screening tool. Shares many strengths and weaknesses of FFQs.	- Useful for estimating dietary patterns over short periods. - Low cost. - Low participant and researcher burden. - Relatively simple coding.	- Generally, very short, cannot determine total intakes. This is of concern for microbial research as determination of effects are limited. - Cross-cultural/cross-country comparisons are limited unless diets are comparable. - Restricted to items that are listed in the instrument.
**Retrospective and prospective**		
**Diet histories**—combination of multiple methods, typically 24-h recalls, food frequency questionnaires, and food diaries. More applicable in clinical settings by experienced dieticians to generate an in-depth analysis at an individual level.	- Long periods > 1 month can determine habitual intake. - Combinations of methods is ideally suited to capture accurate dietary intake during a period of interest surrounding faecal matter collection. - Facilitates assessment of meal patterns and food preparation. - Typically uses automated tools that have been adapted for self-administration.	- No standardised protocols available. - Meal based approaches is not suitable for individuals with irregular eating patterns. - High participant and researcher burden. - Requires complex analytical methods. - Expensive, as requires experienced interviewer and researcher to code data.
**Novel technologies**—collect and process dietary data using wearable hardware (such as sensors) and software (such as web-based programmes and mobile apps based on traditional dietary assessment tools). Many have close agreement to traditional methods, yet noticeable differences persist when comparing against the gold standard, doubly labelled water techniques [92].	- Facilitates real-time data entry and results irrespective of location. - Enhanced portion size quantitation and food waste estimating using digitally captured photos. - Reduces participant burden and increases motivation (dependent on participants’ technological ability). - Facilitates easier prompting to reduce mis-recording. - Automation of web-based recording reduces the burden on researchers and interviewers.	- Due to novelty, no validation performed to determine the quality of the technology. - Prone to similar measurement errors as traditional assessment tools. - Potential security risk using a web-based computer or mobile-technologies. - Requires participant education/training if the tool is not intuitive. - Potential high initial costs of specialist equipment and software.

This table is adapted from Nutritools [89]

#### Bias, error, and further limitations

Besides collection methodology, the majority of nutrition research investigates diet as a limited series of macro and micronutrients [96] covered by food composition databases [19]. Dietary mediators of gut microbial action may not be described, overlooking the impact of > 26,000 biochemicals encapsulated within the food matrix, as well as the production, preparation, and consumption of foods with the potential passage of environmental and food-borne microbial communities [16, 19]. Zhang and Li recently demonstrated that consuming cooked foods drastically reduced microbial diversity in comparison with consuming non-thermally processed foods in an animal model [97]. Moving towards detailed descriptions of foods is required, including food processing, cooking methods, and mealtime [76, 98] together with rhythmicity of nutritional responses of nutrient sensing and cellular decision-making [71]. Certain biochemicals have been observed to mitigate or exacerbate the health effects of others present in other foods [19]. For instance, trimethylamine N-oxide (TMAO), a product of trimethylamine (TMA) transformation by the liver, has been extensively associated with the negative health effects linked to red meat consumption [99]. Yet, allicin, a biochemical in garlic, blocks the microbial generation of TMA in the gut, preventing the potential adverse effects of TMAO [19]. Many other bioactive compounds found in food are separately documented elsewhere within the literature; however, extensive systematic collaboration is required for a unified database. Barabasi et al. displayed that an advanced library for garlic and cocoa can be developed by integrating machine learning into study searches for aggregation despite numerous diverse sources [19, 100]. Future efforts could utilise this technology to efficiently analyse large datasets to develop global databases for the benefit of researchers and institutes in the food domain [101]. For example, the FiberTAG project is tagging fibre types, including soluble and insoluble dietary fibre and prebiotic oligosaccharides, by measuring biomarkers related to the gut microbiota in order to aid progression in future diet-microbiome research [102].

Although epidemiological research has succeeded in identifying a link between the gut microbiome and nutrients derived from food composition databases [103], the association with specific food sources remains underexplored. Johnson et al. recently highlighted that measuring food intakes may provide increased insight into day-to-day variations in microbiota than a traditional nutrient model [55]. For example, red wine has been shown to be associated with increased microbial alpha-diversity that was not observed with other alcohols hypothesised to be related to the high polyphenol content of red wine [104]. However, the investigators were unable to confirm due to the restricted descriptions of bioactive compounds available.

While many further limitations and biases are worthy of discussion, the final note on this subject should highlight the importance of rigorous reporting to allow for scientific reproducibility. Standardised reporting guidelines of all future research efforts should be developed following a consultative process [105]. Extensions to current guidelines, such as the STROBE-nut, have been successfully implanted [105]. While these are currently only specified within a number of nutrition journals, investigators are encouraged to incorporate the guidance offered despite this.

### Investigating diet-microbiome interactions

The pliable nature of the gut microbiota composition facilitates its modulation via environmental factors, the most important of which is diet [106]. Yet, to date, unscrambling the effects of diet and the gut microbiota on host health has proved challenging; particularly considering the two are closely aligned [103]. Moreover, the presence of highly complex crosstalk between diet, microbiota, and the host has proven a major confounder [4] with full characterisations of the complex interactions between dietary substrates and metabolites, and the crosstalk between host and microbes not yet fully explored [107].

**Fig. 3:**
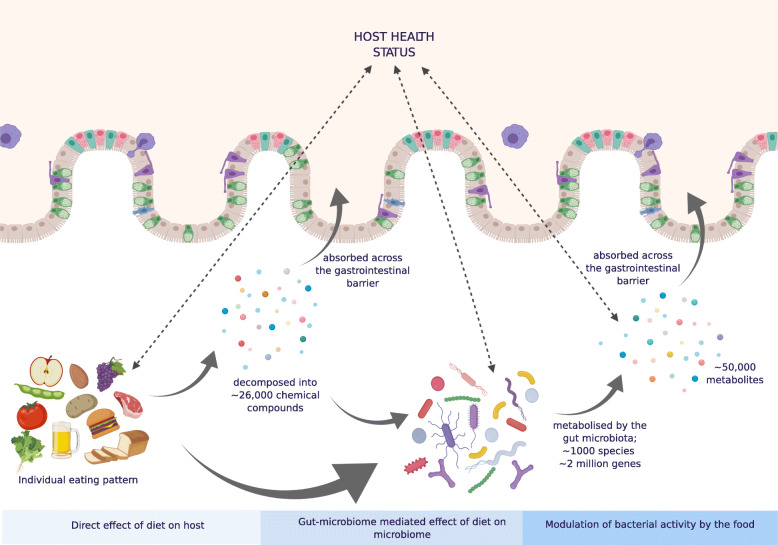
Diet contributes to the intertwined mechanisms between the microbiota and host that have yet to be fully elucidated [107]. The physical structure and chemical composition of dietary intake is a large effector of health; moreover, dietary nutrients that bypass host absorption and secretion support the activity of the gut microbiome [109], yet there remains a complex inter-change between multiple other components outside of these. Image created with Biorender.com

Diet influences not only the microbial composition, but also regulates the activity of the ecosystem (and its effects on the host) without noticeable compositional alterations [108] (Fig. 3). Further investigations to identify factors that influence these three-way interactions between the host, diet and the microbiota are required. These complex and intricate relations demands the employment of a holistic approach moving beyond simple association studies [110]. While diet may at times have minimal impact on the microbial community structure, the production of dietary metabolites may differ [111]. Thousands of dietary biomolecules are present within a food matrix, many of which are unknown [19]. Identification of strains implicated in the metabolism of dietary substrates remains unclear with multiple others performing similar or the same pathways, some of which work synergistically [16]. To date, the wealth of metabolic functionality studies has demonstrated the extensive reach of the gut microbiota throughout the metabolic system of gnotobiotic [112], antibiotic-treated animals [113, 114], and also some human studies [115]. Recent advances in mathematical models to capture key aspects of the gut microbiome and its hosts’ physiological response facilitate the generation of hypotheses that can later be experimentally validated [115]. The future inclusion of data points in artificial intelligence (AI) models beyond the typical may also further assist in expanding our understanding of diet-microbiome relations, for example, the functional genomic analyses of carbohydrate utilisation of strains and species and the cross-feeding of fermentation products and vitamins unidirectionally or bidirectionally [116, 117]. For instance, the growth of many butyrate-producing gut bacteria, such as *F. prausnitzii*, *S. variabile*, and *Roseburia*, has been shown to be auxotrophic for B vitamins, and therefore rely on exogenous sources [117], with links between increased consumption of B vitamins and abundance of taxa [118]. By including such data, a greater understanding of diet-based approaches to modulate beneficial microbes and improve health may be modelled [117, 119].

Reconceptualization of the current approaches towards big data technical methodologies and improved study design may assist in further characterisation of the diet-microbial landscape [120]. Technological advances in high-throughput -omic technologies have greatly improved the accessibility to functional information surrounding the microbiome [49]. Such studies are indispensable for the progression of the field alongside the increased focus on developing comprehensive and reproducible workflows and improved choice of methods and scientific rigour in the conduct of the study [59]. Integrating the fields of microbiology, genetics, epigenetics, metabolomics, proteomics, and nutrition, we can consolidate our understanding of techniques, thereby develop investigations which may capture a richer and more coherent picture [121]. For example, in a 2019 study, investigators explored diet-microbiome relations and their individual impact on visceral fat mass (VFM). The pair-wise association and conditional analyses, together with machine learning approaches, enabled to both estimate and separate the effects of diet and the gut microbial community on host VFM [103]. Additionally, the integration of multiple fields assists in overcoming some of the limitations of individual technologies by looking at a broader picture of disease networks rather than, for example, compounds in isolation [122]. Furthermore, the emergence of novel visualisation tools such as bio-orthogonal click chemistry labelling [123] and optical windows for real-time tracking has shown potential but has yet to be fully applied [49]. Consideration must be made as to how to best integrate quantitative imaging techniques with the quantitative pipeline to advance diagnostics, improve population health through disease prevention and management [49].

Longitudinal large multi-centre studies are required which employ standardised protocols for the collection of validated biomarkers of health for phenotyping, subject demographics, dietary information, biological samples, laboratory processing, genetic analyses, and data analysis and manipulation [3]. Generation of substantial data pools could be overcome by integration of the mechanistic, hypothesis-driven approach with machine learning AI [124]. Machine learning methods to identify microbiota characteristics associated with host phenotypes of interest can be categorised into two types, supervised and unsupervised learning [125]. Supervised learning can be useful when aiming to predict a health outcome or a phenotype based on microbiome profiles. It also enables the formation of a prediction model based on the multitudes of microbial taxa, enabling a view of the ecosystem rather than organisms in isolation. Unsupervised learning can also assist in identifying patterns within the ecosystem as well as within a population, as demonstrated by the concept of enterotypes [125]. Although currently in its infancy within the microbiome field, AI-based recommendation systems (RS) have shown promise [126]. By integrating blood parameters, dietary patterns, anthropometrics, physical activity, and gut microbiota into a RS, Zeevi et al. were able to predict glycaemic response to meals [127]. The researchers successfully manipulated dietary intake to alter the gut microbiome, enabling them to reduce host postprandial glucose response [127]. However, RS are limited by our current incomplete understanding of microbial metabolic pathways, microbial community, and definition of a healthy microbiome.

Large databanks typically include limited phenotypes to limit researcher burden and cost, whereas typically small finite samples facilitate more in-depth phenotyping [128]. Maximising phenotypic trait data within a substantial sample size allows for cohorts to be sufficiently powered to discover and replicate associations [129]. Meta-analysis techniques can then be used to pool samples or to combine with clinical trial results in order to detect ‘true’ signals and to reduce false-positive rates, strengthening the findings by demonstrating reproducibility [130]. By providing data from varying perspectives, researchers are able to answer a diverse array of scientific questions, whereby findings are more generalisable [128]. For example, AI technologies, such as those mentioned above, have been shown to outperform humans in predicting patient re-admission following congestive heart failure [131], though these technologies alone cannot provide translatable information for human health, requiring integration of AI, interventions, and mechanistic studies.

## Conclusions and outlook

To advance our understanding of the role of diet-microbiota interactions on human health and disease, it is crucial to step back and re-evaluate current approaches. Standardisation and optimisation of methodologies may assist in capturing the complex spectrum of these relations. To inform dietary strategies for the prevention and amelioration of chronic metabolic disease states, we first need to ensure intrinsic data is sufficient and relevant, in a manner that considers the deeply individual aspects of both diet and microbiome. Characterisation of the natural intricacies of the ecosystem and the interactions existing between its multiple members at various levels of complexity remains critical. Both micro- and macro-scale influences that drive microbial variation should be considered, from spatial organisation to transmission amongst hosts and between the host and the environment. A comprehensive, multidisciplinary research agenda is required to accurately describe the gut microbial composition and function. Individual and combined complexities of both microbial research and nutrition research demand reconsideration of standard approaches, with a push towards gold standard protocols, further emphasis on the use of randomised control trials, and mechanistic studies, and analysis techniques that include big data, multi-omics, and machine learning approaches. Without multifactorial approaches towards investigations of the diverse aspects of the microbiome, diet, and diet-microbiome relations, we will be limited in our progression towards therapeutic interventions on a personalised or population level. While the path ahead may be unclear in how we may reach these targeted strategies to improve host health, the approaches outlined within this review may assist in a collaborative move forward.

## Data Availability

Not applicable

## Abbreviations

FMT: Faecal microbiome transplantation
TMAO: Trimethylamine N-oxide
TMA: Trimethylamine
VFM: Visceral fat mass
RS: Recommendation systems
AI: Artificial intelligence
